# The relationship between speed and curvature differs in autistic and non-autistic tracing movements

**DOI:** 10.1038/s41598-026-37067-z

**Published:** 2026-02-15

**Authors:** Jennifer L. Cook, Dagmar S. Fraser, Lydia J. Hickman, Rebecca Brewer, Dongsung Huh

**Affiliations:** 1 School of Psychology and Centre for Human Brain Health, University of Birmingham, Edgbaston, Birmingham, B15 2TT UK; 2 MRC Cognition and Brain Sciences Unit, University of Cambridge, Cambridge, CB2 7EF UK; 3 School of Psychology, Royal Holloway University of London, London, TW20 0EX UK; 4 MIT-IBM Watson AI Lab, Cambridge, MA 02142 USA

**Keywords:** Autism, Kinematics, Motor function, One third power law, Minimum-jerk, Neuroscience, Physics, Psychology, Psychology

## Abstract

A growing field documents differences in autism in movement-based tasks such as handwriting, throwing a ball and social gestures. Usefully, complex movements such as social gestures and cursive handwriting can be decomposed to signature regularities that match those of “pure frequency” shapes including spirals, ellipses and rounded triangles. By studying the way that autistic and non-autistic people draw pure frequency shapes we can therefore predict movement patterns for a range of complex functional actions and gestures, and we can gain insights that may help us understand potential mechanisms underlying any differences. Correspondingly, we recorded the x and y position of a stylus tip as 21 autistic and 19 non-autistic adults (matched for age, IQ, and sex) traced a range of pure frequency shapes on a tablet. The relationship between speed and curvature across the pure frequency shapes typically follows a set of mathematical equations which can be thought of as a “spectrum of power laws”. We compared the speed-curvature relationship between groups and, additionally used fast Fourier transform (FFT) to investigate potential mechanisms. Autistic and non-autistic adults differed in the relationship between speed and curvature. FFT revealed that non-autistic participants narrowly modulated speed oscillations around target frequencies, while autistic participants showed broader speed modulation profiles, potentially indicative of differences in the bodily filtering of outgoing movement signals coming from the brain. Our results enable predictions about how autistic individuals might execute a range of functional movements, and may help in the development of support structures for complex tasks like writing.

**Supplementary Information:**

The online version contains supplementary material available at 10.1038/s41598-026-37067-z.

## Introduction

Research into autism spectrum conditions (referred to as autism hereafter in line with the majority preference expressed in surveys of the autistic community^1–4^) has classically focused on social cognition, language and communication. A burgeoning field, however, has characterised autistic bodily movement and motor control profiles^5–10^. Studies have suggested that autistic individuals typical move differently when carrying out both ‘fine’ motor tasks such as handwriting^11–14^, as well as ‘gross’ motor tasks such as riding a bike or throwing a ball^15^. A recent review reported an estimated 50–88% of autistic children experience motor challenges on standardized motor assessments and/or functional questionnaires^16^. Motor control differences in autism have also been linked to social communication outcomes^7,17–22^. It remains unclear whether there are commonalities in autistic movement that could feasibly underpin differences across a range of actions. That is, one’s hand must follow a very different trajectory when gesturing to a conversation partner, compared to writing in cursive script, at present it is unclear whether there are features of autistic movement that have the potential to predict movement patterns across these two, very different, tasks.

Studies of motor control suggest that complex movements, once decomposed, consist of common signatures; thus, by studying simple movements it is possible to gain insight into more complex action sequences^23^. Our previous work revealed differences in the kinematics of arm movements in autism^24^. We found that, for simple arm swing movements, non-autistic individuals accelerate and decelerate gradually, producing minimum-jerk kinematic profiles^25,26^, by contrast autistic individuals accelerate and decelerate rapidly, producing movements characterised by increased jerk (change in acceleration). We subsequently observed similar results in an independent sample^20^ and in our studies of facial movements^27,28^, and other research teams have reported increased jerk in arm^29,30^, leg^31^, neck^32^, and head^33^ movements in autistic adults and/or children. Extant work therefore reliably demonstrates that, compared to non-autistic individuals, autistic individuals move with different kinematic profiles characterised by increased jerk (change in acceleration).

**Fig. 1:**
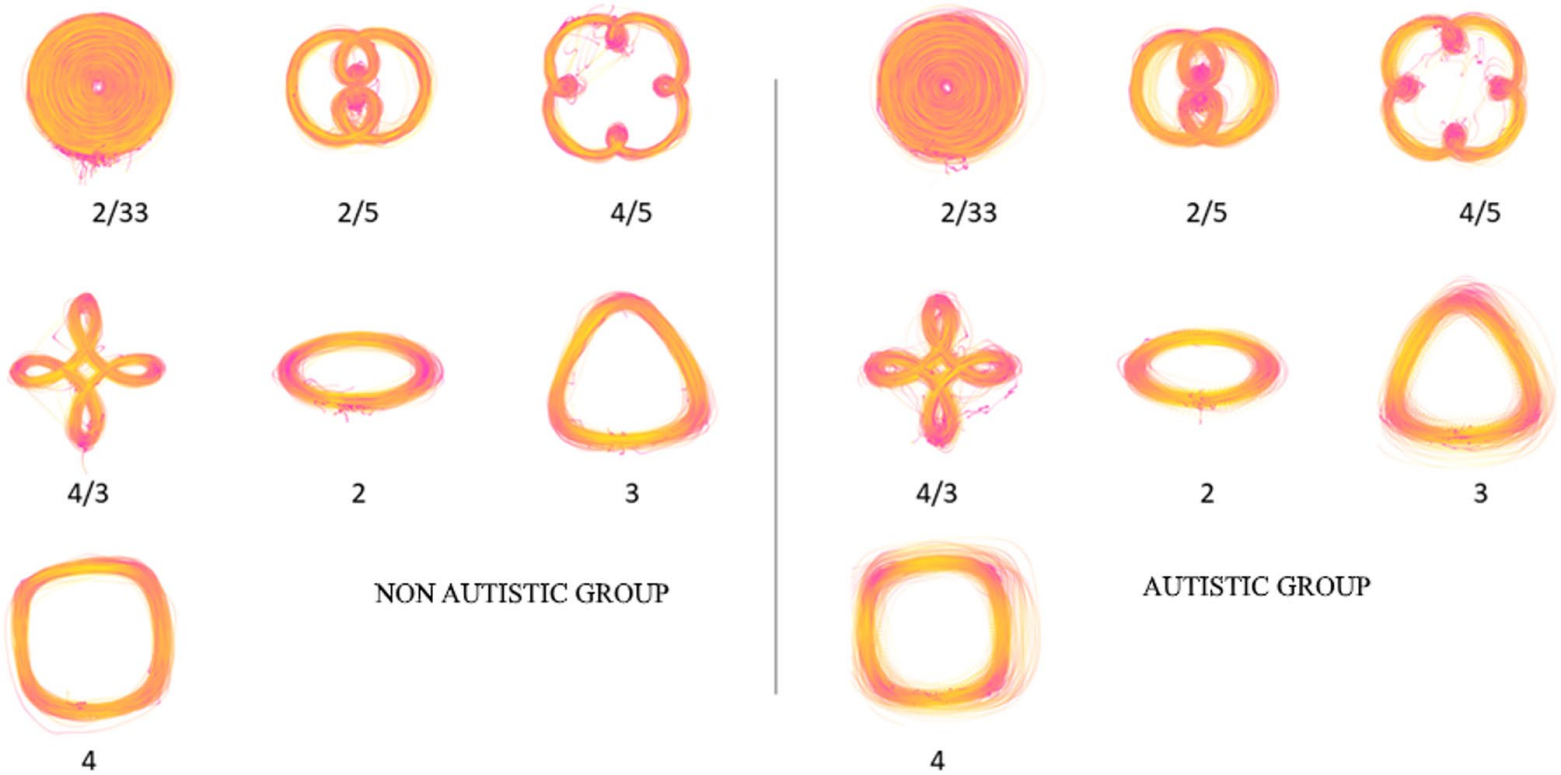
Raw movement trajectory data. Raw trajectory data for all participants in the non-autistic (*left*) and autistic (*right*) groups, for all trials. The colour scheme represents speed from dark pink (low speed) to yellow (high speed) for spiral (angular frequency (AF) = 2/33), double-nested (AF = 2/5), single-nested (AF = 4/5), petalled (AF = 4/3), ellipse (AF = 2), rounded triangle (AF = 3) and rounded square (AF = 4) shapes. Note that more tightly curved trajectories tend to be represented as dark pink (i.e., low speed).

Autistic individuals may move with increased jerk because their movement profiles differ with respect to what are often called ‘power laws’ in the motor control literature. Finding out whether this is indeed the case would pave the way for a better understanding of mechanisms underpinning differences in autistic movements and might assist in the development of support structures for functional movement-based tasks. One of these laws, referred to as the one third power law - also known as the two-third power law relating angular speed and curvature^34^ - describes the relationship between tangential movement speed and trajectory curvature. When freehand drawing or producing an ellipse, for example, people move slowly around the “corners” compared to straighter segments^35–40^. The one third power law states that there is a negative relationship between (log) curvature and (log) speed (Fig. 1) - as curvature increases, speed decreases - and that the gradient of this relationship is -1/3 (hence giving the power law its name).

Understanding whether, and how, autistic movement differs from these power laws might help us to predict and understand differences in autistic movement across a whole range of useful actions. Irrespective of whether one is gesturing during conversation, swiping across the screen on a smartphone, aimlessly doodling, or writing cursive script, movements typically follow power laws. Work by Huh and Sejnowski^36^ showed that the speed profile that characterises functional movements (like doodles) can be decomposed and represented as ‘density’ (i.e., energy) within different frequency bands that are characterised by different ‘pure frequency shapes’ including spirals, rounded-squares and -triangles (Fig. 1). However, the research that fed into the development of power laws has not put neurodiversity at the forefront. Thus, we do not know how applicable these power laws are to autistic people. It is possible that autistic people tend to draw pure frequency shapes in a different fashion. Knowing this would enable us to make predictions about how autistic people might execute a whole host of movements including swiping, doodling and handwriting. Such predictions would be useful in building support structures to help with functional tasks such as handwriting and may guide us towards a better understanding of underlying mechanisms.

The one third power law (described above) is the most famous of the power laws, but more recent work has shown that it belongs to a spectrum of power laws. The spectrum of power laws observes that the gradient of the relationship between speed and curvature is not always -1/3 but can take a range of values determined by the shape of the movement trajectory, more specifically by the ‘angular frequency’ of the shape. The angular frequency is defined as the number of curvature oscillations per cycle. An ellipse has an angular frequency of two because in every 360° (or 2π radians) cycle of angular displacement (θ), there are two points where the curvature of the trajectory changes dramatically, at the two “corners” of the ellipse. Thus, for an ellipse, plotting curvature against a cycle of θ reveals two peaks. A rounded square has an angular frequency of four, corresponding to four changes in curvature in every cycle. A logarithmic spiral has an angular frequency approaching zero because in every cycle there is only near constant trajectory curvature^36,41^. That is, the gradient of the speed-curvature relationship is steep for shapes, like spirals, with low angular frequency and shallow for shapes, like rounded squares, with high angular frequency (Fig. 1). Power laws have been observed in a range of human movements including hand drawings^34,42^, smooth pursuit eye movements^43^, speech^44^ and walking^45^. They have also been demonstrated across species^46^ - including humans^36,47,48^, non-human primates^49,50^, *drosophila* larvae^40^, the elephant^51^ and the bumblebee *Bombus terrestris audax*^52^.

Since autistic movements tend to be characterised by increased jerk it is possible that they differ across the entire spectrum of power laws. Extant studies^53,54^, however, have focused on ellipses (angular frequency = 2), and have therefore not assessed whether autistic and non-autistic movements differ across the full angular frequency spectrum. Thus, we know only that elliptical movement tends to be different, but we know little about the other ‘pure frequency’ shapes. This means that we cannot make predictions for how autistic movement differs in complex, fluid actions like doodling, handwriting or gestures because we only have data for a single shape and complex movement is a composite across the full range of pure frequency shapes. Importantly, high jerk movements do not *necessarily* result from an atypical power law relationship between speed and curvature. Increased jerk may also be a consequence of decomposing shapes into a greater number of submovements^55–57^ and/or from moving particularly quickly or slowly^58,59^. Since the number of submovements is theoretically independent of speed-curvature gradient, it is possible that although autistic individuals exhibit kinematics with increased jerk they, nevertheless, follow non-autistic power laws. Consequently, whether autistic and non-autistic movements differ across the full angular frequency spectrum must be empirically tested.

Understanding whether autistic movements differ from non-autistic movements across the spectrum of power laws may help us move towards an understanding of underlying mechanisms. Deviations from power laws may indicate differences in motor cortical control policies (i.e., how the brain plans and executes movement speed;^25,60^ and/or biomechanical constraints (i.e., how the body executes movement^47^). In contrast, simply moving at a different speed, in the absence of violations of power laws, may point to other mechanisms. The speed of one’s movement is thought to be controlled by a subjective cost function that balances the gains from moving against the energy expenditure costs of doing so, thus, one might move particularly fast if one’s brain was up-weighting the value of the gains or down-playing the energetic costs^57^. Likewise, decomposing movements into a greater number of submovements (without violating power laws) could be a consequence of differences in perceptual processing styles. That is, if one perceived a movement to comprise of multiple piecemeal actions – as suggested in previous work^61^ - one might break down that movement into a series of submovements. To progress towards an understanding of the mechanisms underpinning motor control in autism, we must assess whether movements differ across the spectrum of power laws that typically characterise the relationship between speed and curvature.

We recorded x and y position at 133 Hz while autistic and non-autistic adults traced, on a tablet device, a range of pure frequency shapes that included a spiral (angular frequency (AF) = 2/33), a shape with double-nested loops (AF = 2/5), a shape with single-nested loops (AF = 4/5), a petalled shape (AF = 4/3), an ellipse (AF = 2), a rounded triangle (AF = 3) and a rounded square (AF = 4). To investigate systematic differences in the relationship between speed and curvature we calculated the gradient of the relationship between (log) speed and (log) curvature for each shape and compared gradients between the groups. We predicted that we would observe a main effect of group such that speed-curvature gradients would be significantly steeper for autistic compared to non-autistic participants thus indicating movement that is characterised by greater speed changes between the straights (fast sections) and the corners (slower sections). We anticipated that this difference between the groups would be present across the entire spectrum of pure frequency shapes. To gain insight into potential mechanisms underpinning any differences we also used the fast Fourier transform to explore amplitude spectral density across all angular frequencies.

## Results

### Autistic and non-autistic movements differ across the spectrum of power law speed-curvature gradients

**Fig. 2:**
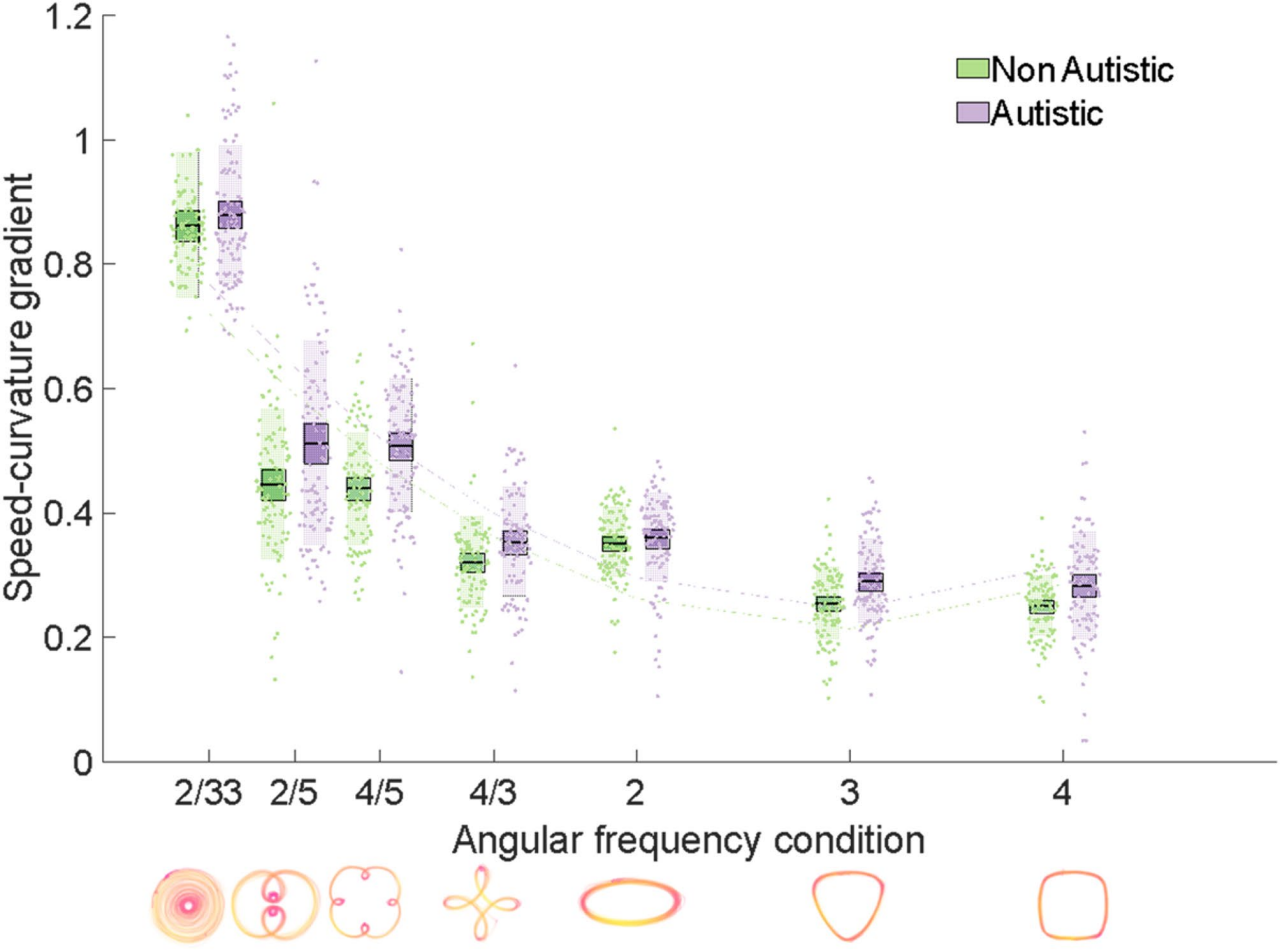
A graph depicting speed-curvature gradients for autistic and non-autistic groups. Speed-curvature gradients plotted against angular frequency, for autistic and non-autistic groups. Speed-curvature gradients were significantly negatively related to angular frequency for both autistic and non-autistic groups. However, a main effect of group indicated that speed-curvature gradients were significantly higher for the autistic relative to the non-autistic group, across all levels of angular frequency. Bars = mean, box = standard error of the mean (SEM), individual data points plotted, second order polynomial line of best fit plotted for illustration purposes.

For speed-curvature gradients, an ANOVA conducted on model coefficients from our mixed model (speed-curvature gradient ~ Shape + Group + (Shape x Group) + (1|Trial) + (1|Participant)); revealed that there was a main effect of group (*F*(1,1285) = 5.29, *p* = .021), main effect of shape (*F*(6,1285) = 1206.40, *p* < .001) and group x shape interaction (*F*(6,1285) = 2.62, *p* = .016; Fig. 2). As can be seen from Table 1 the grand mean is 0.44 (intercept), the estimated mean for the autistic group is 0.46 (grand mean + autistic beta = 0.44 + 0.02), and the estimated mean for the non-autistic group is 0.42 (grand mean + non-autistic beta = 0.44−0.02). The main effect of group thus indicates that autistic participants exhibited steeper speed-curvature gradients than non-autistic participants. The main effect of shape indicates that speed-curvature gradients decreased as a function of angular frequency; compared to the grand mean, low angular frequency shapes were characterised by steeper gradients (positive *β* estimates in Table 1) and higher angular frequency shapes were characterised by shallower gradients (negative *β* estimates in Table 1).

The interaction between group and shape denotes the extra change in the estimated mean gradient over and above the main effects. Table 1, for instance, indicates that non-autistic participants for the shape with double-nested loops (AF = 2/5) have an estimated mean gradient of 0.45 (i.e., the sum of the following *β* coefficients: intercept + non-autistic + double-nested + non-autistic double-nested = 0.44 − 0.02 + 0.04 − 0.01 = 0.45). In contrast, the estimated mean gradient for the autistic group for the double-nested shape is 0.51 (intercept + autistic + double-nested + autistic double-nested = 0.44 + 0.02 + 0.04 + 0.01 = 0.51; a difference between the groups of 0.06). *β* coefficients relating to the interaction between group and shape illustrate that the mean estimated difference between groups in speed-curvature gradients varies as a function of shape. Notably, this interaction term is statistically significant for double-nested, single-nested and ellipse shapes. For double-nested and single-nested shapes, the difference between the groups (i.e., higher gradient in the autistic than non-autistic group) is greater than the difference that would be predicted from the main effects of group and shape alone (see Fig. 2). For the ellipse, the difference between the groups is smaller than the difference that would be predicted from the main effects alone.

**Table 1:** Model parameters for speed-curvature gradient mixed model.

	β estimate	SE	t statistic	DoF	*p* value	Lower CI	Upper CI
Speed-curvature gradient							
Intercept	0.44	0.01	51.05	1285	< 0.001***	0.42	0.45
Non-autistic	− 0.02	0.01	− 2.30	1285	0.022*	− 0.04	0.00
Autistic	0.02	0.01	2.30	1285	0.022*	0.00	0.04
Spiral (2/33)	0.43	0.01	78.65	1285	< 0.001***	0.42	0.44
Double-nested (2/5)	0.04	0.01	7.30	1285	< 0.001***	0.03	0.05
Single-nested (4/5)	0.04	0.01	6.28	1285	< 0.001***	0.02	0.05
Petalled (4/3)	− 0.10	0.01	− 17.64	1285	< 0.001***	− 0.11	− 0.09
Ellipse (2)	− 0.08	0.01	− 14.02	1285	< 0.001***	− 0.09	− 0.07
Triangle (3)	− 0.16	0.01	− 29.03	1285	< 0.001***	− 0.17	− 0.15
Square (4)	− 0.17	0.01	− 29.42	1285	< 0.001***	− 0.18	− 0.16
Non-autistic (NA) Spiral	0.01	0.01	1.95	1285	0.052	0.00	0.02
NA double-nested (2/5)	− 0.01	0.01	− 2.32	1285	0.020*	− 0.02	0.00
NA single-nested (4/5)	− 0.01	0.01	− 2.00	1285	0.046*	− 0.02	0.00
NA petalled (4/3)	0.01	0.01	0.34	1285	0.737	− 0.01	0.02
NA ellipse (2)	0.01	0.01	2.22	1285	0.027*	0.00	0.02
NA triangle (3)	0.00	0.01	− 0.24	1285	0.809	− 0.01	0.01
NA square (4)	0.00	0.01	0.05	1285	0.967	− 0.01	0.01

Note. Model equation: Speed-curvature gradient ~ Shape + Group + (Shape x Group) + (1|Trial) + (1|Participant). R^2^ ordinary = 0.864 (0.810 without random effects), R^2^ adjusted = 0.863 (0.808 without random effects). Statistics as produced by the MATLAB fitlme function. *p < .05, **p < .01, ***p < .001. SE = standard error, DoF = degrees of freedom, CI = confidence interval.

The theoretical values for speed-curvature gradients are as follows: spiral = 0.664, double-nested = 0.652, single-nested = 0.533, petalled = 0.433, ellipse = 0.355, triangle = 0.260, square = 0.199. An important, unpredicted, observation is that values for both groups differ from the theoretical values for speed-curvature gradients (autistic t(651) = 7.45, *p* < .001; non-autistic t(646) = 10.40, *p* < .001).

Further exploratory mixed models (Supp. Mat. 3) confirmed that groups differed in the gradient of the relationship between speed and curvature, and not in the fit of the regression line and revealed that the groups differed in terms of minimum and maximum speed values, but not curvature values. These post hoc analyses demonstrate that differences in speed-curvature gradients between autistic and non-autistic groups are not due to drawing more or less curved movement trajectories (i.e., they are not spatial differences) but rather are due to reaching higher maximum speeds, and slowing to lower minimum speeds.

### FFT of speed offers insight into mechanisms underpinning high speed-curvature gradients in autism

**Fig. 3:**
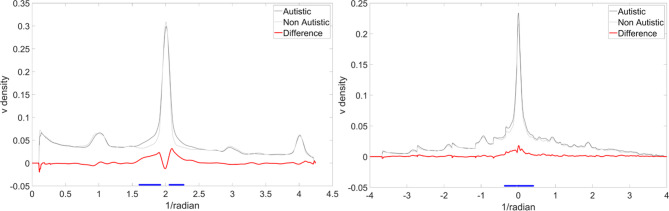
Amplitude spectral density graphs. Left panel: Illustrative density spectrum for angular frequency two. Note the clear peak at two, which is more precise (narrower peak flanks) for the non-autistic group. Right panel: Amplitude spectral density of velocity for all angular frequency-defined conditions aligned to zero. Black line = autistic, Grey line = non-autistic, Red line = autistic - non-autistic, blue lines denote bootstrapped clusters of statistical significance.

A continuous drawing of an ellipse typically has a clearly defined density peak in a band centred around angular frequency two (because there are two peaks in curvature in an ellipse) and low density in other bands (see Fig. 3 left panel for example amplitude spectral density function for the ellipse shape). We employed FFT to explore a potential post hoc explanation for the atypically high speed-curvature gradients in the autistic group (see above). One possibility is that autistic participants were modulating their speed in a way that is appropriate for drawing a tighter corner (requiring a greater deceleration and hence greater jerk). For instance, if participants modulated speed in a way that is appropriate for drawing a rounded triangle’s corners when actually drawing a square this would result in an abnormally high speed-curvature gradient (because speed-curvature gradient increases with decreasing angular frequency) and would be revealed by a leftward-shifted peak in the FFT-derived amplitude spectral density function. To explore this potential post hoc interpretation, we calculated amplitude spectral density functions for all participants, for all angular frequency-defined shapes. Visual exploration of the data clearly showed that amplitude spectra were not leftward-shifted for the autistic group; in fact, peaks were almost exactly aligned. Nevertheless, we observed that peaks were narrower for non-autistic relative to autistic participants. To illustrate this, we aligned amplitude spectra along the target frequency (Fig. 3 right panel) such that the zero point corresponds to an angular frequency of two for drawings of elliptical shapes, three for the triangle shape, four for the square shape and so on, and used bootstrapped t-tests at all 1001 points on the angular frequency spectrum.

This analysis revealed that autistic participants exhibited more variable (see the broader width of the peak in Fig. 3) speed oscillations around the target frequency. Bootstrapped *t*-tests (1000 boots) showed two significant clusters of difference - defined as clusters of difference that occurred in less than 5% of comparisons with resampled distributions (highlighted in blue in Fig. 3 right panel) - between the autistic and non-autistic groups. These clusters fell either side of the target frequency, thus statistically confirming the narrowness of the speed oscillation profile around the target frequency for the non-autistic, relative to autistic, group. That is, there is an increase of amplitude spectral density away from the target angular frequency in the autistic group. It should be noted that this analysis was repeated for curvature and no significant differences were observed thus suggesting that differences lie in the precision of modulation of speed not curvature (Supp. Mat. 4).

**Fig. 4:**
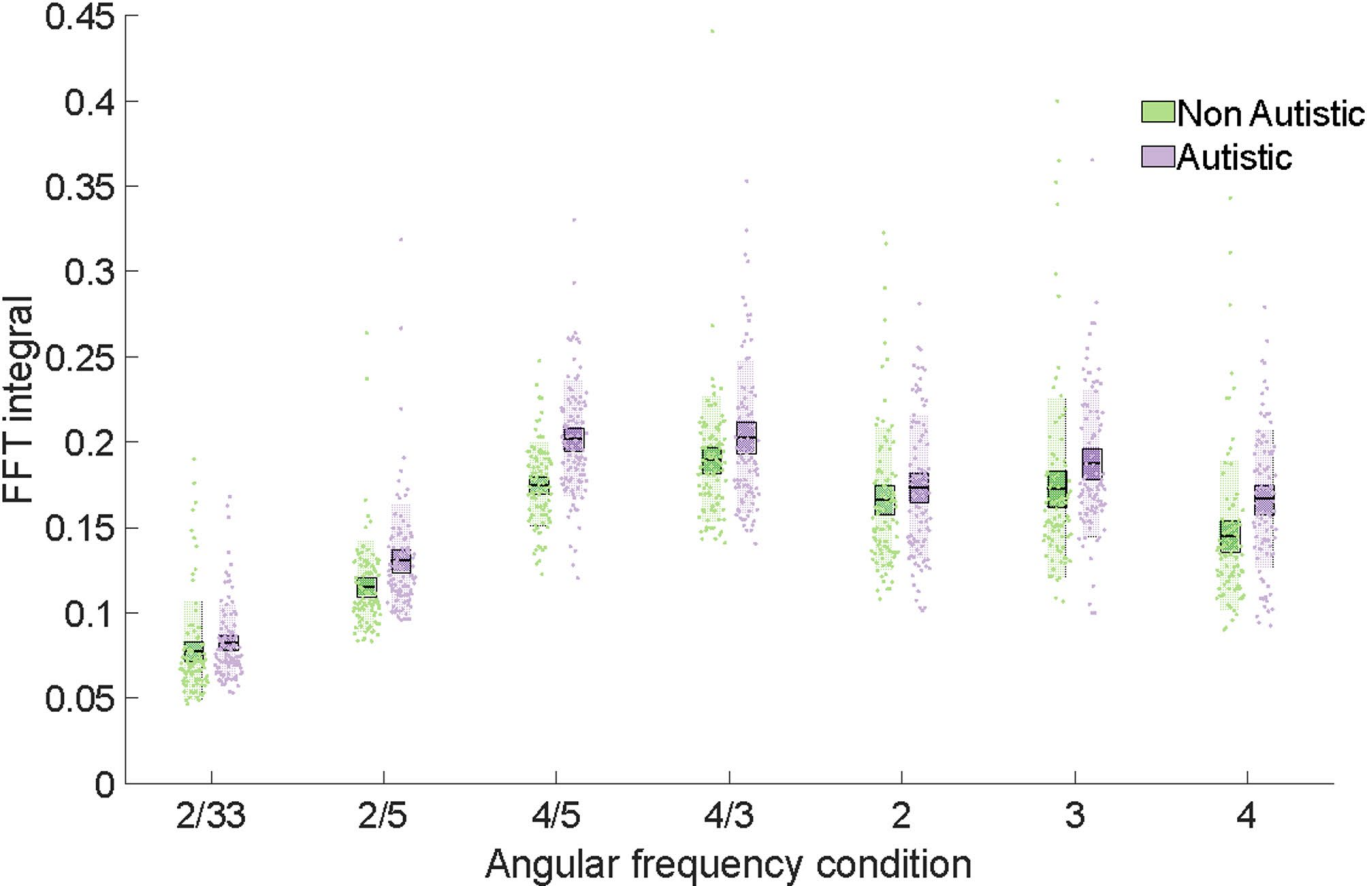
A graph depicting the FFT integrals for autistic and non-autistic groups. FFT integral against angular frequency for autistic and non-autistic groups. FFT integral values were significantly higher for the autistic relative to the non-autistic group and this difference was particularly large for the 4/5 condition. Bars = mean, box = SEM, individual data points plotted.

To further validate this, and account for repeated observations from the same participants across multiple trials, we used trapezoidal numerical integration (MATLAB trapz.m) to calculate the area under the amplitude spectral density function (FFT integral) for all trials, for all participants. FFT integrals were submitted to an effects-coded, mixed effects model with shape, group and the interaction between shape and group as fixed effects and trial and participant as random effects (FFT integral ~ Shape + Group + (Shape x Group) + (1|Trial) + (1|Participant); Table 2). An ANOVA conducted on model coefficients revealed that there was a significant main effect of group (*F*(1,1283) = 4.63, *p* = .032), main effect of shape (*F*(6,1283) = 357.19, *p* < .001) and group x shape interaction (*F*(6,1283) = 2.52, *p* = .020). The main effect of group indicates that the FFT integral was greater for autistic relative to non-autistic participants and the interaction demonstrates that this difference varied as a function of angular frequency, with the difference being small for the spiral and particularly large for the rounded-square. In sum, we observed that non-autistic participants showed narrow and tall peaks around target frequencies whereas density spectra for autistic participants were broader and shorter (Fig. 4).

**Table 2:** Model parameters for FFT integral mixed model.

	β estimate	SE	t statistic	DoF	*p* value	Lower CI	Upper CI
FFT integral							
Intercept	0.16	0.00	42.40	1283	< 0.001***	0.15	0.16
Non-autistic	− 0.01	0.00	− 2.15	1283	0.032*	− 0.02	0.00
Autistic	0.01	0.00	2.15	1283	0.032*	0.00	0.02
Spiral (2/33)	− 0.08	0.00	− 37.69	1283	< 0.001***	− 0.08	− 0.07
Double-nested (2/5)	− 0.03	0.00	− 16.77	1283	< 0.001***	− 0.04	− 0.03
Single-nested (4/5)	0.03	0.00	16.30	1283	< 0.001***	0.03	0.04
Petalled (4/3)	0.04	0.00	18.96	1283	< 0.001***	0.04	0.04
Ellipse (2)	0.01	0.00	6.68	1283	< 0.001***	0.01	0.02
Triangle (3)	0.02	0.00	11.52	1283	< 0.001***	0.02	0.03
Square (4)	0.00	0.00	− 0.12	1283	0.908	0.00	0.00
Non-autistic (NA) Spiral	0.01	0.00	2.91	1283	0.004**	0.00	0.01
NA Double-nested (2/5)	0.00	0.00	− 0.11	1283	0.912	0.00	0.00
NA Single-nested (4/5)	0.00	0.00	− 1.66	1283	0.097	− 0.01	0.00
NA Petalled (4/3)	0.00	0.00	0.05	1283	0.959	0.00	0.00
NA Ellipse (2)	0.00	0.00	1.41	1283	0.160	0.00	0.01
NA Triangle (3)	0.00	0.00	− 0.50	1283	0.620	− 0.01	0.00
NA Square (4)	0.00	0.00	− 2.03	1283	0.043*	− 0.01	0.00

Note. Model equation: FFT integral ~ Shape + Group + (Shape x Group) + (1|Trial) + (1|Participant). R^2^ ordinary = 0.693 (0.528 without random effects), R^2^ adjusted = 0.690 (0.523 without random effects). Statistics as produced by the MATLAB fitlme function. *p < .05, **p < .01, ***p < .001. SE = standard error, DoF = degrees of freedom, CI = confidence interval.

## Discussion

The current study compared tracing movements executed by autistic and non-autistic adults to investigate potential differences in the power law relationship linking movement speed and curvature. Compared to age-, IQ- and sex-matched non-autistic participants, the gradient of the relationship between speed and curvature was significantly higher for autistic individuals. This was seen across multiple pure frequency shapes. The difference between the groups was particularly large for the shapes defined by the angular frequencies 2/5 (double-nested shape) and 4/5 (single-nested shape). Post hoc analyses demonstrated that differences in speed-curvature gradients between the groups were not due to spatial differences; autistic participants did not draw more/less curved trajectories. Instead, gradient differences were due to differences in speed modulation. That is, autistic participants’ movement was characterised by accelerating to higher maximum speeds (on the straights) and slowing to lower minimum speeds (at the corners).

An important observation is that both autistic and non-autistic values deviate from theoretical predictions of speed-curvature gradients (as calculated by Huh and Sejnowski^36)^. This finding aligns with recent work questioning the universality and uniformity of power laws^62^. It has often been assumed that human and non-human movement invariantly adheres to power laws. That is, speed-curvature gradients have been referred to as ‘kinematic regularities’ or ‘motor invariances’, and have been said to characterise all biological movement. However, our recent review of the literature^62^ revealed considerable variability in the actual value of speed-curvature gradients across studies and highlighted a number of cases where speed-curvature gradients do not align with theoretical predictions. Such variability can arise from multiple sources, including differences in equipment or data processing methods. These factors may explain why the values here observed differ from the theoretical values. Nevertheless, in the present study, autistic and non-autistic participants used identical equipment, and all data were processed using the same analysis pipeline, thus these factors cannot explain differences *between* the groups.

Higher speed-curvature gradients for autistic compared to non-autistic individuals may indicate underlying differences in motor control policies and/or biomechanical constraints. It has been theorised that smooth movement – that accords with the power law spectrum - may be a higher-order goal of the human motor system^25,60,63^. That is, our brains may plan how to move in a minimally jerky fashion. According to this view, departures from the power law spectrum indicate brain-based differences in motor cortical control policies. Alternative accounts argue that the power law spectrum is a product of the biomechanics of the body^47,48^. That is, muscles and joints effectively comprise a system of filters that produce power law compliant motion. In other words, our brain does not necessarily plan how to move smoothly but our body acts as a filter system such than even noisy movements end up smooth. According to this view, departures from the power law relationship may indicate differences in biomechanical constraints – e.g., differences in muscle stiffness^64,65^ or strength^66^. However, the current study is not designed to arbitrate between central and peripheral nervous system explanations for differences in movement kinematics. Furthermore, within the central nervous system it is thought there are different systems for planning versus execution of movements^67^, thus, even if one could establish that the current results are due to differences in motor control policies in autism further studies would be needed to explore whether differences relate to the planning of smooth action or its execution. Nevertheless, the higher speed-curvature gradients that we here observe open important new questions about whether there tend to be differences in the biomechanics of autistic muscles, limbs and joints and/or whether autistic movements tend to be governed by different brain-based policies.

Although the current results cannot arbitrate between neural and biomechanical mechanisms, our FFT analysis emphasises the importance of biomechanics. FFT decomposes the speed profile and effectively represents it as energy that indicates speed changes in different angular frequency bands. This method enables us to visualise how precisely a participant is executing the relevant movements: for example, tracings of ellipses should, in theory, be dominated by energy in the angular frequency *two* band (because an ellipse has *two* dramatic changes in speed – one at each “corner”). We observed that, whilst non-autistic participants showed narrow peaks around target frequencies, speed profiles for autistic participants’ tracings were less narrow. Post hoc interpretation of this finding may be informed by auditory science: The FFT operation that we here carried out on the speed wave is analogous to the operation that is carried out on sound waves by the basilar membrane in the human ear. The basilar membrane behaves as if it contains a bank of overlapping bandpass filters (‘auditory filters’) that decompose sound waves into their constituent frequency components. Each filter is tuned to a particular centre frequency, with that basilar membrane filter responding maximally to that frequency and progressively less to more distal frequencies. The relative response of the filter, as a function of frequency, is known as the auditory filter shape and is analogous to the amplitude spectral density function that we calculated for each angular frequency-defined shape. In normal hearing participants auditory filters are ‘narrow’, they are sharply tuned around the centre frequency^68^. However, less narrow auditory filters are reported in autistic individuals^69^, wherein the bandwidth of the filter was significantly wider than bandwidths measured in non-autistic individuals^70^. Plaisted and colleagues suggest that their results indicate differences in the peripheral filtering of incoming sensory signals, as opposed to indicating differences in central mechanisms (though they note that the two are not mutually exclusive). Plaisted and colleagues’ interpretation is highly relevant for our results. The appropriate modulation of speed as a function of curvature has been argued to, at least in part, comprise the end product of a series of filters^47,71^, which can tune a noisy motor signal to the appropriate target (angular) frequency. Thus, mirroring Plaisted and colleagues’ results, which may indicate differences in the filtering of *incoming* sensory signals, our results may indicate differences in the filtering of *outgoing* motor signals. Since motor signals are hypothetically filtered by our muscles and joints^47,71^ this hypothesis is in line with studies reporting differences in muscle stiffness, joint mobility^64,65^ and muscle strength^66^ in autistic people. Clearly, further studies are required to empirically test this hypothesis, and arbitrate between models that postulate differences in the filtering of motor signals in autism and those that postulate different (e.g., more noisy) motor signals per se^33^.

A further potential source of group differences in speed–curvature gradients concerns the interpretation of task instructions. Movements are typically smoother and less jerky when participants draw fluidly and automatically^72^ rather than focusing on producing the ‘correct’ shape and making small corrective adjustments. To promote fluidity, each trial began with written instructions encouraging participants to draw the shapes continuously without correction and an experimenter oversaw practice trials. We cannot, however, unequivocally rule out the possibility that autistic participants were more biased toward accuracy than non-autistic participants. Nevertheless, jerky movements of the head, neck, face, and in gait cycles have been reported in autism—movements that do not have a ‘correct’ form. This pattern lends support to the view that the present results reflect a general movement style in autism, rather than a task-specific strategy.

The current results may also have practical implications for autism screening and diagnosis. Tools that can discriminate between autistic and non-autistic individuals from an early age are much sought after^73–75^ and movement-based methods are particularly appealing because they do not rely on language and can therefore be used with non-speaking children^76^. Here we showed that differences between autistic and non-autistic groups are particularly large for the double- and single-nested shapes, thus suggesting that the speed-curvature gradient relating to these particular trajectories may be usefully incorporated into automated tools^37^. Since speed-curvature gradients are mean speed- and scale- invariant^36^ they have the added advantage that they are robust against differences in speed and size that are known to affect autistic movement, but which do not systematically discriminate between autistic and non-autistic individuals (i.e., handwriting analysis has linked autism to both micro- and macro-graphia ^12,13,77^).

For these findings to translate into clinically meaningful tools, further work is required. First, the current study provides an in-depth analysis of a small sample, which precludes the assessment of potential subgroup effects. It remains to be established, for example, whether the speed–curvature gradients observed here differ between autistic and non-autistic individuals of different genders or ethnic backgrounds. Such extensions are essential because existing screening tools have particularly low precision for girls and non-white children^78^, leading to disparities in access to, and further delays in autism diagnoses for these groups^79–82^. In other work, we have observed differences in facial movement that persist across subgroups^27,28^; it is important to establish whether the same holds for tracing movements. Second, the present results cannot determine whether analogous movement differences are present early in development. It cannot be assumed that patterns observed in adulthood directly reflect those seen in childhood, since trajectories for motor development cannot be assumed to be linear. As autism is most commonly diagnosed in early childhood, it will be crucial to establish whether differences in speed–curvature coupling are evident at this stage. A further consideration concerns task design. The paradigm employed here relies on verbal and written instruction, which would limit its use with minimally-speaking populations. Future work should therefore adapt the task for use with minimally-speaking participants. Finally, it will be important to determine the specificity of the observed effects. Future studies should test whether atypical speed–curvature gradients can discriminate between autism and other developmental motor conditions, such as developmental coordination disorder, to ensure that any diagnostic application of these measures is both sensitive and specific.

An important question concerns the potential applied relevance of our findings. By asking our participants to draw shapes that span the angular frequency spectrum, we have been able to show that autistic individuals exhibit different speed-curvature gradients. In principle, the shapes we asked participants to draw can be combined to create an infinite number of trajectory shapes. Conversely any trajectory (even a seemingly random doodle) can be decomposed into density across the angular frequency spectrum^36,41^. Previous studies, however, only compared autistic and non-autistic participants with respect to elliptical trajectories. Our results suggest that the kinematics of autistic tracing movements differ across the whole angular frequency spectrum, with pronounced differences for the double- and single-nested shapes. Furthermore, autistic participants’ movement was characterised by accelerating to higher maximum speeds (on the straights) and slowing to lower minimum speeds (at the corners). In practical terms these results suggest that autistic individuals might exhibit more rapid changes from accelerating to decelerating when executing a range of everyday movements including social gestures, cursive handwriting and throwing a ball, and that this might be particularly evident when these movements are looped (as with the double- and single-looped shapes). The consequences of this might be increased energy expenditure - potentially making movements feel more tiring - and/or less accurate movements (since speed modulation is generally linked to accuracy). In line with this, previous studies have shown that minimally-jerky movement, that aligns with the power law spectrum, is important across a range of functional motor tasks including handwriting^83,84^ and throwing^25,26,85^, and that autistic individuals often experience difficulties with such tasks^11–15^. It is possible that the differences in the speed-curvature relationship that we have observed underpin these functional challenges. Ongoing endeavours to train movement kinematics^86^ have the potential to reveal novel methods which may be used to support autistic people in performing functional motor tasks. Nevertheless, these speculative predictions are yet to be empirically verified.

## Summary

To conclude, our results evidence that the kinematics of tracings differ between autistic and non-autistic adults such that autistic adults tend to accelerate to higher maximum speeds (on the straights) and slow to lower minimum speeds (at the corners). Post hoc exploratory analyses raise the possibility that this may be due to differences in how selectively autistic bodies filter outgoing movement signals as evidenced by an FFT analysis showing increased variation in speed oscillations around the target frequency. These data raise important questions about motor control policies and biomechanical constraints that govern autistic movement and may have important implications for identification and support systems for autistic people.

## Methods

### Sample size rationale

The sample size was based on a previous result in which we observed a significant difference in jerk (t(27) = 3.28, *P* = .003, Cohen’s d = 1.26) with 15 non-autistic participants and 14 autistic participants (Cook et al., 2013). Using GLIMMPSE (Kreidler et al., 2013) we calculated that 6 participants are necessary in each group to have 99% power to detect an equivalent effect. To guard against the “winners curse” (Button et al., 2013) we planned to recruit at least triple the recommended sample size.

### Participants

Data were collected from 21 autistic adult participants and 19 non-autistic adult participants. All participants in the autistic group had a clinical diagnosis from an independent clinician. In addition, participants in the autistic group completed the Autism Diagnostic Observation Schedule-2^87,88^ with a trained administrator and reached the criteria for autism or autism spectrum. There were no significant differences between the groups in terms of age, gender or IQ (as measured by the Wechsler Adult Intelligence Scale^89^ for the autistic group and the Wechsler Abbreviated Scale of Intelligence^90^ for the non-autistic group). The autistic group had significantly higher Autism Quotient (AQ^91^ scores than the non-autistic group (see Table 3)). Informed consent was obtained from all participants prior to data collection. All experimental procedures were approved by the University of Birmingham Research Ethics Committee (ERN_12-0971P) and performed in accordance with the Declaration of Helsinki.

**Table 3:** Group demographics.

	Autistic	Non-autistic	Group comparison statistics
N	21	19	
Age mean (SD)	36.10(11.414)	30.37(9.418)	t(38) = 1.720, *p* = .094
Gender (M: F)	19:2	18:1	χ^2^(1,40) = 0.261, *p* = .609
IQ mean (SD)	109.52(14.059)	108.37(14.781)	t(38) = 0.190, *p* = .801
AQ mean (SD)	35.10(8.746)	18.94(6.485)	t(37) = 6.455, *p* < .001

Note. There were no differences between the autistic and non-autistic groups in terms of age, gender, Intelligence Quotient (IQ) or Autism Quotient (AQ). SD = standard deviation, M:F = male to female ratio.

### Procedure

A commercial tablet device (Wacom Cintiq 22HD, sampling rate 133 Hz, accuracy +/-0.5 mm) was used to record movement trajectories. Participants traced shapes displayed on the screen with a stylus digitizer in their dominant hand. There were seven conditions relating to seven shapes with different angular frequencies (Fig. 1). Each trial commenced with written instructions. Participants placed the stylus near the centre of the screen, on a red arrow for spiral shape, or at the darkest part of a blue line for shapes. All movements were anticlockwise, and participants were encouraged to draw the shapes fluidly without making corrections. Trials began when participants initiated drawing movements. A trial finished after participants had continuously traced the shape’s perimeter producing curvature peaks equal to the angular frequency of the shape multiplied by ten. Participants could choose to repeat, or abandon and restart, a trial in which they felt fluidity was not achieved. Trials with discontinuous data (e.g., clockwise movement, removals of the stylus) were rejected during the experiment. The first five successfully completed trials were retained for each of the seven shapes.

### Data analysis

All data were processed and analysed in MATLAB R2022a. The data and code required to reproduce results and figures is freely available at (https://osf.io/j4ncd/overview?view_only=db95e4126bd14a9bb5aa5401f81d8e7c).

### Task compliance

Deviation values (i.e., deviation from the ‘correct’ shape) for each trial were calculated as the absolute mean of the normal distance to the tangent of the nearest point on the shape’s curve^92^. Speed-curvature gradient values (see below for calculation) may be highly sensitive to production errors (deviations from the ideal shape and the corresponding corrective movement). Thus, if autistic and non-autistic individuals differ in the magnitude of deviation this could create an illusory difference in speed-curvature gradients. A linear mixed model predicting error with group (fixed effect) and with trial number and participant ID as random effects demonstrated no significant differences between autistic and non-autistic participants in terms of drawing deviations (F(1, 1297) = 2.30, *p* = .129). Therefore, task compliance was judged to be equivalent between the two groups.

### Calculation of speed-curvature gradients

**Fig. 5:**
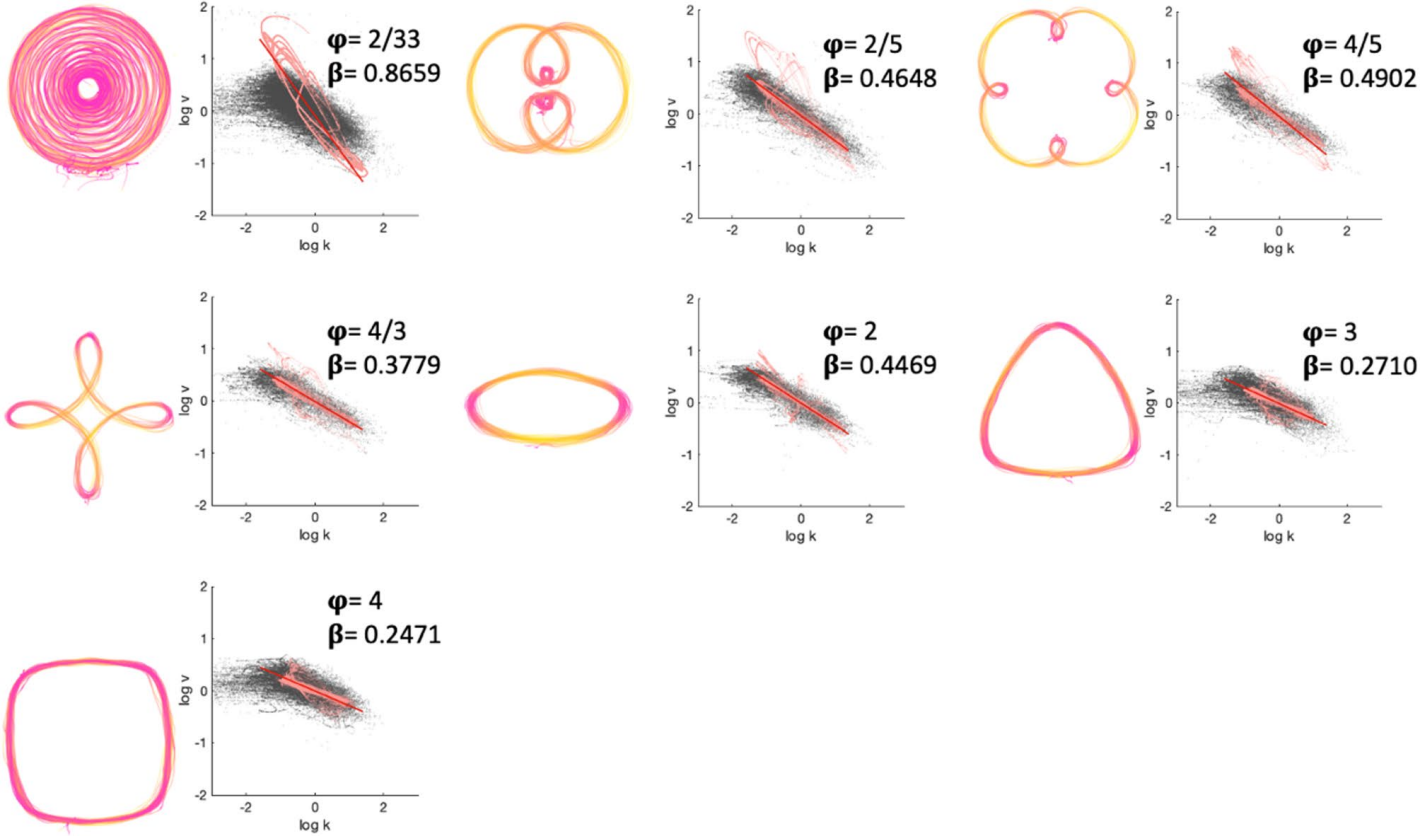
Typical movement examples from a single participant. The colour scheme represents speed from dark pink (low speed) to yellow (high speed). Log speed vs. log curvature is shown to the right of each shape. Grey dots: Raw curvature-speed data. Pink dots: Bandpass filtered curvature-speed data. Red line: Line of best fit. Measured $\:\beta\:$ exponent is given in the inset for each shape.

Our dependent variable was the gradient of the relationship between (log) speed and (log) curvature (i.e., the speed-curvature gradient) given the absolute value of $\:\beta\:$ in Eq. 1. See Supp. Mat. 1 for further details. A higher $\:\beta\:$ means a steeper slope of the log curvature vs. log speed relationship (see Fig. 5 for example for single participant raw data and corresponding $\:\beta\:$ estimates). For non-autistic participants, there should be a negative relationship between angular frequency $\:\phi\:$ of shape and $\:\beta\:$ value (Huh & Sejnowski, 2015).

1 $$\:v\left(\theta\:\right)\:\alpha\:\:\kappa\:{\left(\theta\:\right)}^{-\beta\:},\:\:\:\:\:\:\:\:\:\:\:\:\:\:\:\left(\beta\:\left(\phi\:\right)=\frac{2}{3}\:\left(\frac{1+\frac{{\phi\:}^{2}}{2}}{1+{\phi\:}^{2}+\:\frac{{\phi\:}^{4}}{15}}\right)\right)$$

An effects-coded, mixed effects model was fitted (using the *fitlme* function of the Statistics and Machine Learning Toolbox in MATLAB) with speed-curvature gradient (absolute value) as the dependent variable (DV) and group (autistic, non-autistic), shape (angular frequency-defined shape 2/33, 2/5, 4/5, 4/3, 2, 3, 4), and the interaction between shape and group as fixed effects. Group and shape were specified as categorical predictors. A random intercept for trial number (1, 2, 3, 4, 5) was fitted to account for practice or fatigue effects (e.g., speeding up as a function of trial) and a random intercept for participant (defined as a categorical predictor) was included. To obtain *F* and *p* values we employed analysis of variance for linear mixed effects models using the MATLAB ANOVA function.

### Frequency analysis

The speed profile that characterises a continuous drawing movement can be thought of as a complex wave. As with other complex waves - like sound waves - one can use Fourier transform to decompose the wave and represent it as density within different frequency bands. A high-pitched sound wave, for example, would have high spectral density in high frequency bands. Similarly, a drawing of a high angular frequency shape such as a square would have a density peak in the band around an angular frequency of four and low density in other bands. Thus, Fourier transform of the speed wave allows one to quantify the relative spectral density of different angular frequencies that characterise a particular drawing.

To plot amplitude spectral density as a function of angular frequency, (log) speed was examined. The peaks’ periodicity, in every 2$\:\pi\:$ radians, allow the determination of angular frequencies present in the movements of the stylus. Asymmetrical fast Fourier transform (FFT) was employed, which returned the amplitude spectral density of all angular frequencies. To avoid spectral leakage when presenting a finite length signal, such as the (log) speed, to the FFT; the first and last values of the signal were made less discontinuous by removing a second order polynomial trend. Angular frequency densities returned by the FFT were then interpolated to obtain uniformly sampled values at 1001 arbitrary points.

### Exploratory analyses of speed, jerk and submovements

For further exploratory analyses we calculated jerk, speed and number of submovements (see Supp. Mat. 2).

## Supplementary Information

Below is the link to the electronic supplementary material.

Supplementary Material 1

## Data Availability

Data and analysis are available online at the following repository (https:/osf.io/j4ncd/overview?view_only=db95e4126bd14a9bb5aa5401f81d8e7c).

## References

[1] Bottema-Beutel, K., Kapp, S. K., Lester, J. N., Sasson, N. J. & Hand, B. N. Avoiding ableist language: suggestions for autism researchers. *Autism Adulthood*. **3** (1), 18–29 (2021). 36601265 10.1089/aut.2020.0014 PMC8992888

[2] Bury, S. M., Jellett, R., Spoor, J. R. & Hedley, D. It defines who I am or it’s something I have: what Language do [Autistic] Australian adults [on the autism Spectrum] prefer? *J. Autism Dev. Disord.* **53** (2), 677–687 (2023). 32112234 10.1007/s10803-020-04425-3

[3] Keating, C. et al. Autism-related Language preferences of English-speaking individuals across the globe: A mixed methods investigation. *Autism Res.* **16**(2) (2023). 10.1002/aur.2864 PMC10946540 36474364

[4] Kenny, L. et al. Which terms should be used to describe autism? Perspectives from the UK autism community. *Autism: Int. J. Res. Pract.* **20** (4), 442–462 (2016). 10.1177/1362361315588200 26134030

[5] Fournier, K. A. et al. Decreased static and dynamic postural control in children with autism spectrum disorders. *Gait Posture*. **32**, 6–9 (2010). 20400311 10.1016/j.gaitpost.2010.02.007 PMC2919314

[6] Gowen, E. & Hamilton, A. Motor abilities in autism: a review using a computational context. *J. Autism Dev. Disord*. **43**, 323–344 (2013). 22723127 10.1007/s10803-012-1574-0

[7] Leary, M. R. & Hill, D. A. Moving on: autism and movement disturbance. *Ment Retard.* **34**, 39–53 (1996). 8822025

[8] London, E. B. Categorical diagnosis: a fatal flaw for autism research? *Trends Neurosci.* **37**, 683–686 (2014). 25465942 10.1016/j.tins.2014.10.003

[9] Stoit, A. M. B., van Schie, H. T., Slaats-Willemse, D. I. E. & Buitelaar, J. K. Grasping motor impairments in autism: not action planning but movement execution is deficient. *J. Autism Dev. Disord*. **43**, 2793–2806 (2013). 23619948 10.1007/s10803-013-1825-8

[10] Zampella, C. J., Haley, L. W. L. A., Hutchinson, M. & Ashley, M. A. G. Motor skill differences in autism spectrum disorder: a clinically focused review. *Curr. Psychiatry Rep.* **23**, (2021). 10.1007/s11920-021-01280-6 34387753

[11] Beversdorf, D. Q. et al. Brief report: macrographia in high-functioning adults with autism spectrum disorder. *J. Autism Dev. Disord*. **31**, 97–101 (2001). 11439759 10.1023/a:1005622031943

[12] Godde, A., Tsao, R., Gepner, B. & Tardif, C. Characteristics of handwriting quality and speed in adults with autism spectrum disorders. *Res. Autism Spectr. Disord*. **46**, 19–28 (2018).

[13] Grace, N., Enticott, P. G., Johnson, B. P. & Rinehart, N. J. Do handwriting difficulties correlate with core Symptomology, motor proficiency and attentional behaviours? *J. Autism Dev. Disord*. **47**, 1006–1017 (2017). 28083779 10.1007/s10803-016-3019-7

[14] Rosenblum, S., Ben-Simhon, A., Meyer, H., Gal, E. & S. & Predictors of handwriting performance among children with autism spectrum disorder. *Res. Autism Spectr. Disord*. **60**, 16–24 (2019).

[15] Staples, K. L. & Reid, G. Fundamental movement skills and autism spectrum disorders. *J. Autism Dev. Disord*. **40**, 209–217 (2010). 19685284 10.1007/s10803-009-0854-9

[16] Kangarani-Farahani, M., Malik, M. A. & Zwicker, J. G. Motor impairments in children with autism spectrum disorder: A systematic review and Meta-analysis. *J. Autism Dev. Disord*. **54**, 1977–1997 (2024). 36949273 10.1007/s10803-023-05948-1

[17] Bhat, A. N. Motor impairment increases in children with autism spectrum disorder as a function of social Communication, cognitive and functional impairment, repetitive behavior Severity, and comorbid diagnoses: A SPARK study report. *Autism Res.* **14**, 202–219 (2021). 33300285 10.1002/aur.2453 PMC8176850

[18] Cook, J. From movement kinematics to social cognition: the case of autism. *Phil Trans. R Soc. B*. **371**, 20150372 (2016). 27069049 10.1098/rstb.2015.0372 PMC4843610

[19] Donnellan, A. M., Hill, D. A. & Leary, M. R. Rethinking autism: implications of sensory and movement differences for Understanding and support. *Front. Integr. Neurosci.* **6**, 124 (2012). 23372546 10.3389/fnint.2012.00124 PMC3556589

[20] Edey, R. et al. Interaction takes two: typical adults exhibit mind-blindness towards those with autism spectrum disorder. *J. Abnorm. Psychol.* **125**, 879–885 (2016). 27583766 10.1037/abn0000199

[21] McNaughton, K. A. & Redcay, E. Interpersonal synchrony in autism. *Curr. Psychiatry Rep.* **22**, 12 (2020). 32025922 10.1007/s11920-020-1135-8

[22] Wang, L. A. L., Petrulla, V., Zampella, C. J., Waller, R. & Schultz, R. T. Gross motor impairment and its relation to social skills in autism spectrum disorder: A systematic review and two meta-analyses. *Psychol. Bull.* **148**, 273–300 (2022). 35511567 10.1037/bul0000358 PMC9894569

[23] Yazdani, M., Gamble, G. & Henderson, G. Hecht-Nielsen, R. A simple control policy for achieving minimum jerk trajectories. *Neural Netw.* **27**, 74–80 (2012). 22137550 10.1016/j.neunet.2011.11.005

[24] Cook, J., Blakemore, S. & Press, C. Atypical basic movement kinematics in autism spectrum conditions. *Brain J. Neurol.* **136**, 2816–2824 (2013). 10.1093/brain/awt208 PMC4017873 23983031

[25] Flash, T. & Hogan, N. The coordination of arm movements: an experimentally confirmed mathematical model. *J. Neurosci.* **5**, 1688–1703 (1985). 4020415 10.1523/JNEUROSCI.05-07-01688.1985 PMC6565116

[26] Todorov, E. & Jordan, M. I. Smoothness maximization along a predefined path accurately predicts the speed profiles of complex arm movements. *J. Neurophysiol.* **80**, 696–714 (1998). 9705462 10.1152/jn.1998.80.2.696

[27] Keating, C. T., Sowden-Carvalho, S., O’Donoghue, H. & Cook, J. Mismatching expressions: Spatiotemporal and kinematic differences in autistic and non-autistic facial expressions. *Autism Res. ***Online ahead of print,**10.1002/aur.70157 (2026). 10.1002/aur.70157 PMC12948747 41548888

[28] Keating, C. T. & Cook, J. Facial movements as biomarkers for autism: A bayesian prevalence and machine-learning proof-of-concept study. *Preprint at.*10.31234/osf.io/h4yd7_v1 (2025).

[29] Anzulewicz, A., Sobota, K. & Delafield-Butt, J. T. Toward the autism motor signature: gesture patterns during smart tablet gameplay identify children with autism. *Sci. Rep.* **6**, 31107 (2016). 27553971 10.1038/srep31107 PMC4995518

[30] Yang, H. C., Lee, I. C. & Lee, I. C. Visual feedback and target size effects on Reach-to-Grasp tasks in children with autism. *J. Autism Dev. Disord*. **44**, 3129–3139 (2014). 24974254 10.1007/s10803-014-2165-z

[31] Nobile, M. et al. Further evidence of complex motor dysfunction in drug naïve children with autism using automatic motion analysis of gait. *Autism* **1362361309356929**10.1177/1362361309356929 (2011). 10.1177/1362361309356929 21478224

[32] Miller, H. L. et al. Movement smoothness during dynamic postural control to a static target differs between autistic and neurotypical children. *Gait Posture*. **99**, 76–82 (2023). 36335658 10.1016/j.gaitpost.2022.10.015 PMC10644903

[33] Torres, E. B. & Denisova, K. Motor noise is rich signal in autism research and Pharmacological treatments. *Sci. Rep.* **6**, (2016). 10.1038/srep37422 PMC5116649 27869148

[34] Lacquaniti, F., Terzuolo, C. & Viviani, P. The law relating the kinematic and figural aspects of drawing movements. *Acta Psychol. (Amst)*. **54**, 115–130 (1983). 6666647 10.1016/0001-6918(83)90027-6

[35] Flash, T. & Handzel, A. A. Affine differential geometry analysis of human arm movements. *Biol. Cybern*. **96**, 577–601 (2007). 17406889 10.1007/s00422-007-0145-5 PMC2799626

[36] Huh, D. & Sejnowski, T. J. Spectrum of power laws for curved hand movements. *Proc. Natl. Acad. Sci. U. S. A.* **112**, E3950-3958 (2015). 10.1073/pnas.1510208112 PMC4517202 26150514

[37] Matic, A. & Gomez-Marin, A. A customizable tablet app for hand movement research outside the lab. *J. Neurosci. Methods*. **328**, 108398 (2019). 31412268 10.1016/j.jneumeth.2019.108398

[38] Richardson, M. J. E. & Flash, T. Comparing smooth arm movements with the Two-Thirds power law and the related Segmented-Control hypothesis. *J. Neurosci.* **22**, 8201–8211 (2002). 12223574 10.1523/JNEUROSCI.22-18-08201.2002 PMC6758108

[39] Viviani, P. & Flash, T. Minimum-jerk, two-thirds power law, and isochrony: converging approaches to movement planning. *J. Exp. Psychol. Hum. Percept. Perform.* **21**, 32–53 (1995). 7707032 10.1037//0096-1523.21.1.32

[40] Zago, M., Matic, A., Flash, T., Gomez-Marin, A. & Lacquaniti, F. The speed-curvature power law of movements: a reappraisal. *Exp. Brain Res.* **236**, 69–82 (2018). 29071361 10.1007/s00221-017-5108-z

[41] Huh, D. The Vector Space of Convex Curves: How to Mix Shapes. *ArXiv150607515 Cs Math Q-Bio* (2015). http://arxiv.org/abs/1506.07515.

[42] Wann, J., Nimmo-Smith, I. & Wing, A. M. Relation between velocity and curvature in movement: equivalence and divergence between a power law and a minimum-jerk model. *J. Exp. Psychol. Hum. Percept. Perform.* **14**, 622–637 (1988). 2974873 10.1037//0096-1523.14.4.622

[43] de’Sperati, C. & Viviani, P. The relationship between curvature and velocity in Two-Dimensional smooth pursuit eye movements. *J. Neurosci.* **17**, 3932–3945 (1997). 9133411 10.1523/JNEUROSCI.17-10-03932.1997 PMC6573701

[44] Kuberski, S. R. & Gafos, A. I. The speed-curvature power law in tongue movements of repetitive speech. *PLOS ONE*. **14**, e0213851 (2019). 30883586 10.1371/journal.pone.0213851 PMC6422270

[45] Ivanenko, Y. P., Grasso, R., Macellari, V. & Lacquaniti, F. Two-thirds power law in human locomotion: role of ground contact forces. *Neuroreport* **13**, 1171–1174 (2002). 12151763 10.1097/00001756-200207020-00020

[46] Flash, T. & Hochner, B. Motor primitives in vertebrates and invertebrates. *Curr. Opin. Neurobiol.* **15**, 660–666 (2005). 16275056 10.1016/j.conb.2005.10.011

[47] Gribble, P. L. & Ostry, D. J. Origins of the power law relation between movement velocity and curvature: modeling the effects of muscle mechanics and limb dynamics. *J. Neurophysiol.* **76**, 2853–2860 (1996). 8930238 10.1152/jn.1996.76.5.2853

[48] Schaal, S. & Sternad, D. Origins and violations of the 2/3 power law in rhythmic three-dimensional arm movements. *Exp. Brain Res.* **136**, 60–72 (2001). 11204414 10.1007/s002210000505

[49] Schwartz, A. B. Direct cortical representation of drawing. *Science* **265**, 540–542 (1994). 8036499 10.1126/science.8036499

[50] Abeles, M. et al. Compositionality in neural control: an interdisciplinary study of scribbling movements in primates. *Front. Comput. Neurosci* **7**, (2013). 10.3389/fncom.2013.00103 PMC3771313 24062679

[51] Dagenais, P., Hensman, S., Haechler, V. & Milinkovitch, M. C. Elephants evolved strategies reducing the Biomechanical complexity of their trunk. *Curr. Biol.* **31**, 4727–4737e4 (2021). 34428468 10.1016/j.cub.2021.08.029

[52] James, L., Davies, T. G. E., Lim, K. S. & Reynolds, A. Do bumblebees have signatures? Demonstrating the existence of a speed-curvature power law in Bombus terrestris locomotion patterns. *PLOS ONE*. **15**, e0226393 (2020). 31940358 10.1371/journal.pone.0226393 PMC6961848

[53] Fourie, E., Lu, S. C., Delafield-Butt, J. & Rivera, S. M. Motor control adherence to the Two-thirds power law differs in autistic development. *J. Autism Dev. Disord*. **55**, 873–890 (2025). 38280136 10.1007/s10803-024-06240-6 PMC11828761

[54] Lu, S. C., Rowe, P., Pollick, F. & Delafield-Butt, J. Kinematic and force control features in autistic adults during curvilinear movements. In *International Society for Autism Research (INSAR) 2023 Annual Meeting*SWE, (2023).

[55] Guigon, E., Chafik, O., Jarrassé, N. & Roby-Brami, A. Experimental and theoretical study of velocity fluctuations during slow movements in humans. *J. Neurophysiol.* **121**, 715–727 (2019). 30649981 10.1152/jn.00576.2018

[56] Lee, D., Port, N. L. & Georgopoulos, A. P. Manual interception of moving targetsII. On-line control of overlapping submovements. *Exp. Brain Res.* **116**, 421–433 (1997). 9372291 10.1007/pl00005770

[57] Shmuelof, L., Krakauer, J. W. & Mazzoni, P. How is a motor skill learned? Change and invariance at the levels of task success and trajectory control. *J. Neurophysiol.* **108**, 578–594 (2012). 22514286 10.1152/jn.00856.2011 PMC3404800

[58] Ambike, S. & Schmiedeler, J. P. Invariant geometric characteristics of Spatial arm motion. *Exp. Brain Res.* **229**, 113–124 (2013). 23771586 10.1007/s00221-013-3599-9

[59] Hernandez, M. E., Ashton-Miller, J. A. & Alexander, N. B. The effect of age, movement direction, and target size on the maximum speed of targeted COP movements in healthy women. *Hum. Mov. Sci.* **31**, 1213–1223 (2012). 22225924 10.1016/j.humov.2011.11.002 PMC3330159

[60] Nelson, W. L. Physical principles for economies of skilled movements. *Biol. Cybern*. **46**, 135–147 (1983). 6838914 10.1007/BF00339982

[61] Booth, R., Charlton, R., Hughes, C. & Happé, F. Disentangling weak coherence and executive dysfunction: planning drawing in autism and attention-deficit/hyperactivity disorder. *Philos. Trans. R Soc. Lond. B Biol. Sci.* **358**, 387–392 (2003). 12639335 10.1098/rstb.2002.1204 PMC1693126

[62] Fraser, D. S., Di Luca, M. & Cook, J. L. Biological kinematics: a detailed review of the velocity-curvature power law calculation. *Exp. Brain Res.* **243**, 107 (2025). 40178611 10.1007/s00221-025-07065-0 PMC11968483

[63] Schwartz, A. B. & Moran, D. W. Motor cortical activity during drawing movements: population representation during lemniscate tracing. *J. Neurophysiol.* **82**, 2705–2718 (1999). 10561439 10.1152/jn.1999.82.5.2705

[64] Eggleston, J. D., Harry, J. R. & Dufek, J. S. Lower extremity joint stiffness during walking distinguishes children with and without autism. *Hum. Mov. Sci.* **62**, 25–33 (2018). 30218847 10.1016/j.humov.2018.09.003 PMC6251740

[65] Luginsland, L. A., Haegele, J. A. & Bennett, H. J. Lower extremity joint stiffness of autistic adolescents during running at dual speeds. *J. Biomech.* **149**, 111478 (2023). 36780731 10.1016/j.jbiomech.2023.111478

[66] Ludyga, S., Pühse, U., Gerber, M. & Mücke, M. Muscle strength and executive function in children and adolescents with autism spectrum disorder. *Autism Res.* **14**, 2555–2563 (2021). 34351051 10.1002/aur.2587 PMC9292567

[67] Glover, S. Separate visual representations in the planning and control of action. *Behav. Brain Sci.* **27**, (2004). 10.1017/s0140525x04000020 15481943

[68] Moore, B. C. & Glasberg, B. R. Auditory filter shapes derived in simultaneous and forward masking. *J. Acoust. Soc. Am.* **70**, 1003–1014 (1981). 7288037 10.1121/1.386950

[69] Plaisted, K., Saksida, L., Alcántara, J. & Weisblatt, E. Towards an Understanding of the mechanisms of weak central coherence effects: experiments in visual configural learning and auditory perception. *Philos. Trans. R Soc. Lond. B Biol. Sci.* **358**, 375–386 (2003). 12639334 10.1098/rstb.2002.1211 PMC1693121

[70] Moore, B. C. J. Distribution of auditory-filter bandwidths at 2 kHz in young normal listeners. *J. Acoust. Soc. Am.* **81**, 1633–1635 (1987). 3584698 10.1121/1.394518

[71] Matić, A. Visuomotor phase-locked loop reproduces elliptic hand trajectories across different rhythms. 07.20.500761 Preprint at (2022). 10.1101/2022.07.20.500761 (2022).

[72] Toner, J., Montero, B. G. & Moran, A. The perils of automaticity. *Rev. Gen. Psychol.* **19**, 431–442 (2015).

[73] Fletcher-Watson, S. et al. Attitudes of the autism community to early autism research. *Autism Int. J. Res. Pract.* **21**, 61–74 (2017). 10.1177/1362361315626577 26975669

[74] Pellicano, E., Dinsmore, A. & Charman, T. What should autism research focus upon? Community views and priorities from the united Kingdom. *Autism* **18**, 756–770 (2014). 24789871 10.1177/1362361314529627 PMC4230972

[75] Rinehart, N. J. et al. An examination of movement kinematics in young people with High-functioning autism and asperger’s disorder: further evidence for a motor planning deficit. *J. Autism Dev. Disord*. **36**, 757–767 (2006). 16865551 10.1007/s10803-006-0118-x PMC2000294

[76] Crippa, A. et al. Use of machine learning to identify children with autism and their motor abnormalities. *J. Autism Dev. Disord*. **45**, 2146–2156 (2015). 25652603 10.1007/s10803-015-2379-8

[77] Cartmill, L., Rodger, S. & Ziviani, J. Handwriting of eight-year-old children with autistic spectrum disorder: an exploration. *J. Occup. Ther. Sch. Early Interv*. **2**, 103–118 (2009).

[78] Guthrie, W. et al. Accuracy of autism screening in a large pediatric network. *Pediatrics* **144**, e20183963 (2019). 31562252 10.1542/peds.2018-3963

[79] Gesi, C. et al. Gender differences in misdiagnosis and delayed diagnosis among adults with autism spectrum disorder with no Language or intellectual disability. *Brain Sci.* **11**, 912 (2021). 34356146 10.3390/brainsci11070912 PMC8306851

[80] Mandell, D. S., Listerud, J., Levy, S. E. & Pinto-martin, J. A. Race differences in the age at diagnosis among Medicaid-Eligible children with autism. *J. Am. Acad. Child. Adolesc. Psychiatry*. **41**, 1447–1453 (2002). 12447031 10.1097/00004583-200212000-00016

[81] Mandell, D. S., Ittenbach, R. F., Levy, S. E. & Pinto-Martin, J. A. Disparities in diagnoses received prior to a diagnosis of autism spectrum disorder. *J. Autism Dev. Disord*. **37**, 1795–1802 (2007). 17160456 10.1007/s10803-006-0314-8 PMC2861330

[82] Russell, G. et al. Time trends in autism diagnosis over 20 years: a UK population-based cohort study. *J. Child. Psychol. Psychiatry*. **63**, 674–682 (2022). 34414570 10.1111/jcpp.13505

[83] Edelman, S. & Flash, T. A model of handwriting. *Biol. Cybern*. **57**, 25–36 (1987). 3620543 10.1007/BF00318713

[84] Viviani, P. & Terzuolo, C. Trajectory determines movement dynamics. *Neuroscience* **7**, 431–437 (1982). 7078732 10.1016/0306-4522(82)90277-9

[85] Abend, W., Bizzi, E. & Morasso, P. Human arm trajectory formation. *Brain* **105**, 331–348 (1982). 7082993 10.1093/brain/105.2.331

[86] Foster, N. C. et al. Facilitating sensorimotor integration via blocked practice underpins imitation learning of atypical biological kinematics in autism spectrum disorder. *Autism* **24**, 1494–1505 (2020). 32168992 10.1177/1362361320908104 PMC7383415

[87] Lord, C. et al. Autism diagnostic observation schedule: a standardized observation of communicative and social behavior. *J. Autism Dev. Disord*. **19**, 185–212 (1989). 2745388 10.1007/BF02211841

[88] Lord, C., Rutter, M. & Le Couteur, A. Autism diagnostic Interview-Revised: a revised version of a diagnostic interview for caregivers of individuals with possible pervasive developmental disorders. *J. Autism Dev. Disord*. **24**, 659–685 (1994). 7814313 10.1007/BF02172145

[89] Wechsler, D. *Wechsler Adult Intelligence Scale - Fourth Edition (WAIS-IV)* 1–3 (San Antonio, 2008).

[90] Wechsler, D. *Wechsler Abbreviated Scale of Intelligence* (The Psychological Corporation, 1999).

[91] Baron-Cohen, S., Wheelwright, S., Skinner, R., Martin, J. & Clubley, E. The autism-spectrum quotient (AQ): evidence from asperger syndrome/high-functioning autism, males and females, scientists and mathematicians. *J. Autism Dev. Disord*. **31**, 5–17 (2001). 11439754 10.1023/a:1005653411471

[92] Madirolas, G., Zaghi-Lara, R. & Gomez-Marin, A. Pérez-Escudero, A. The motor wisdom of the crowd. *J. R Soc. Interface*. **19**, 20220480 (2022). 36195116 10.1098/rsif.2022.0480 PMC9532022

