# Supplementary material for: The relationship between speed and curvature differs in autistic and non-autistic tracing movements
Source: Sci Rep. 2026 Feb 15;16:9175. doi: 10.1038/s41598-026-37067-z (PMC12996617; doi:10.1038/s41598-026-37067-z)

Supplementary Material 1

Analyses of minimum and maximum speed

Exploratory analyses were conducted on the minimum and maximum speed and curvature values, using the mixed model formula: DV ~ Condition + Group + (Condition x Group) + (1|Trial) + (1|Participant). The following transforms were used on the data: log (max velocity), reciprocal (max curvature (see below)), reflect reciprocal (min curvature, min velocity). Analyses revealed that the groups differed in terms of minimum and maximum speed values. For maximum speed there was a significant main effect of condition ($F(6,1285) = 61.85$, $p < .001$) and an interaction between group and condition ($F(6,1285) = 2.68$, $p = .014$), indicating that autistic participants generally reached higher maximum speeds but that the difference between the groups was greatest at low, compared to higher angular frequencies. There was no significant main effect of group ($F(1,1285) = 3.08$, $p = .079$). For minimum speed there was a significant main effect of group ($F(1,1285) = 8.31$, $p = .004$), and condition ($F(6, 1285) = 4.78$, $p < .001$), and no interaction between group and condition ($F(6, 1285) = 0.91$, $p = .483$). Examination of β estimates in **Supplementary Table 1** indicates that autistic participants generally exhibited reduced minimum speeds (i.e., a negative β estimate) compared to non-autistic participants.

In contrast, the groups did not differ in terms of their minimum and maximum curvature values. For maximum curvature, the main effect of group was not significant ($F(1, 1285) = 1.74$, $p = .187$), nor was the interaction between group and condition ($F(6, 1285) = 0.90$, $p = .493$). For minimum curvature, non-significant results were found for both the main effect of group ($F(1, 1285) = 1.52$, $p = .218$) and the interaction between group and condition ($F(6, 1285) = 1.58$, $p = .150$).

Supplementary Table 1 Model Parameters for Maximum and Minimum Speed Mixed Models

	β estimate	SE	t statistic	DoF	p value	Lower CI	Upper CI
							**Maximum Speed**
Intercept	-0.30	0.02	-18.90	1285	<.001***	-0.33	-0.27
Non-autistic	-0.03	0.01	-1.76	1285	.079	-0.05	0.00
Autistic	0.03	0.01	1.76	1285	.079	0.00	0.05
2/33	0.04	0.01	3.23	1285	.001**	0.01	0.06
2/5	0.15	0.01	12.71	1285	<.001***	0.12	0.17
4/5	0.04	0.01	3.69	1285	<.001***	0.02	0.07
4/3	-0.02	0.01	-1.32	1285	.186	-0.04	0.01
2	0.02	0.01	1.86	1285	.063	0.00	0.05
3	-0.06	0.01	-4.67	1285	<.001***	-0.08	-0.03
4	-0.18	0.01	-14.99	1285	<.001***	-0.21	-0.16
Non-autistic 2/33	-0.01	0.01	-1.27	1285	.204	-0.04	0.01
Non-autistic 2/5	-0.01	0.01	-1.01	1285	.311	-0.03	0.01
Non-autistic 4/5	-0.02	0.01	-1.34	1285	.181	-0.04	0.01
Non-autistic 4/3	0.03	0.01	2.29	1285	.022*	0.01	0.05
Non-autistic 2	0.03	0.01	2.65	1285	.008**	0.01	0.06
Non-autistic 3	0.00	0.01	0.13	1285	.899	-0.02	0.03
Non-autistic 4	-0.01	0.01	-1.48	1285	.140	-0.04	0.01
							**Minimum Speed**
Intercept	0.59	0.01	74.31	1285	<.001***	0.58	0.61
Non-autistic	0.02	0.01	2.88	1285	.004**	0.01	0.04
Autistic	-0.02	0.01	-2.88	1285	.004**	-0.04	-0.01
2/33	-0.03	0.01	-2.59	1285	.010**	-0.05	-0.01
2/5	-0.04	0.01	-3.80	1285	<.001***	-0.06	-0.02
4/5	0.00	0.01	0.15	1285	.883	-0.02	0.02
4/3	0.00	0.01	0.44	1285	.661	-0.02	0.03
2	0.03	0.01	2.71	1285	.007**	0.01	0.05
3	0.01	0.01	0.89	1285	.372	-0.01	0.03
4	0.02	0.01	2.05	1285	.041*	0.00	0.04
Non-autistic 2/33	-0.01	0.01	-1.02	1285	.310	-0.03	0.01
Non-autistic 2/5	-0.01	0.01	-0.53	1285	.599	-0.03	0.02
Non-autistic 4/5	0.01	0.01	0.47	1285	.639	-0.02	0.03
Non-autistic 4/3	-0.01	0.01	-1.18	1285	.237	-0.03	0.01
Non-autistic 2	0.01	0.01	1.13	1285	.259	-0.01	0.03
Non-autistic 3	0.00	0.01	-0.37	1285	.715	-0.02	0.02
Non-autistic 4	0.02	0.01	1.45	1285	.149	-0.01	0.04

Note. Model equation: DV ~ Condition + Group + (Condition x Group) + (1|Trial) + (1|Participant). Statistics as produced by the Matlab fitlme function. *p < .05, **p < .01, ***p < .001.

Supplementary Material 2

Exploratory analyses: speed, jerk and submovements

Three separate effects coded linear mixed effects models were fitted (using the *fitglme* function of the Statistics and Machine Learning Toolbox in MATLAB) with jerk, submovements and speed as the DVs. Each model included group (autistic, non-autistic), condition (angular frequency 2/33, 2/5, 4/5, 4/3, 2, 3, 4), and the interaction between condition and group as fixed effects. Group and condition were specified as categorical predictors. A random intercept for trial number (1,2,3,4,5) was fitted to account for practice or fatigue effects (e.g., speeding up as a function of trial) and a random intercept for participant (defined as a categorical predictor) was included. The following transforms were used on the data: log (speed, jerk). Where DVs were not normally distributed (i.e., jerk and speed) a logarithmic link function was specified.

Autistic hand movements are characterised by increased jerk at higher angular frequencies

An ANOVA conducted on coefficients from the mixed effects model (Jerk ~ Condition + Group + (Condition x Group) + (1|Trial) + (1|Participant),'Link', 'log') revealed that there was a main effect of condition ($F(6,1285) = 22.70$, $p < .001$) and an interaction between group and condition ($F(6, 1285) = 18.24$, $p < .001$; **Supplementary Fig. 1**). The main effect of group was not significant ($F(1, 1285) = 3.61$, $p = .058$), though there was a numerical difference between groups. As can be seen from **Supplementary Table 2** the grand mean (log(jerk)) is 5.25 (intercept), the estimated mean for the autistic group is 5.45 (grand mean + autistic beta = 5.25 + 0.2), and the estimated mean for the non-autistic group is 5.05 (grand mean + non-autistic beta = 5.25 - 0.2). Thus, relative to the non-autistic group, the autistic group produced numerically more jerky movements. The main effect of condition indicates that jerk increases as a function of angular frequency condition. That is, relative to the grand mean, estimated jerk is lower for conditions 2/33 (β = -0.17), 2/5 (β = -15), 4/5 (β = -0.1) and higher for conditions 4/3 (β = 0.07), 2 (β = 0.33) and 3 (β = 0.08).

The interaction between group and condition denotes the extra change in the estimated mean jerk over and above the main effects. **Supplementary Table 2** indicates that non-autistic

participants in the 2/33 condition have an estimated mean jerk of 4.98 (i.e., the sum of the following β coefficients: intercept + non-autistic + 2/33 + non-autistic 2/33 = 5.25 - 0.2 - 0.17 + 0.1 = 4.98). In contrast, estimated mean jerk for the autistic group in the 2/33 condition is 5.18 (intercept + autistic + 2/33 + autistic 2/33 = 5.25 + 0.2 - 0.17 - 0.1 = 5.18; a difference between the groups of 0.2). Positive interaction βs in **Supplementary Table 2** indicate that the difference between the autistic and non-autistic groups is smaller than that which would be predicted by the main effects of group and condition alone, negative interaction βs indicate that the difference is greater than would be predicted by group and condition alone. Thus, the interaction indicates that the difference in jerk between the autistic and non-autistic groups is particularly large at higher angular frequencies (angular frequency defined shapes 2,3 and 4). For example, the biggest difference between the groups is for the 2 angular frequency shape where estimated jerk for the non-autistic group = 5.25 - 0.2 + 0.33 - 0.21 = 5.17, whereas estimated jerk for the autistic group = 5.25 + 0.2 + 0.33 + 0.21 = 5.99 (a difference of 0.82).

Supplementary Figure 1 Graphs of Speed, Jerk and Submovements for Autistic and Non-autistic Groups. Log speed and jerk, and submovements plotted against angular frequency (condition) for autistic (purple) and non-autistic (green) groups. Bars = mean, box = SEM, individual data points plotted, second order polynomial line of best fit plotted for illustration purposes.

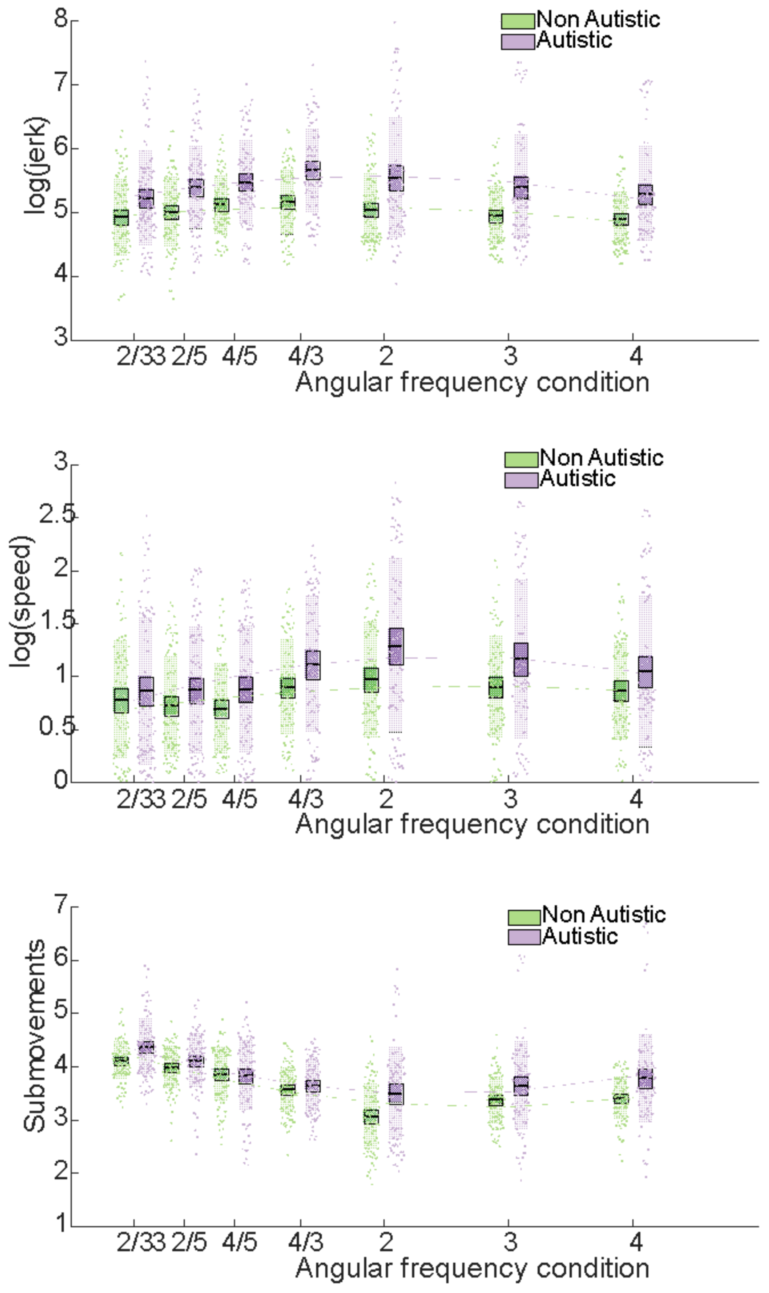

Autistic hand movements are characterised by increased speed at higher angular frequencies

For speed, there was no main effect of Group ($F(1,1285) = 1.16$, $p = .282$). There was, however, a significant main effect of condition ($F(6,1285) = 107.60$, $p < .001$) and group by condition interaction ($F(6,1285) = 18.13$, $p < .001$). Coefficients from a mixed effects model (Speed ~ Condition + Group + (Condition x Group) + (1|Trial) + (1|Participant), 'Link', 'log') can be seen in **Supplementary Table 2**. The main effect of condition was driven by an increase in speed as a function of angular frequency. Notably, the β values (**Supplementary Table 2**) show that, relative to the grand mean, speed is most greatly increased for the angular frequency 2 condition, thus speed and angular frequency are related by an inverted-U-shaped function (**Supplementary Fig. 1**). β coefficients relating to the interaction between group and condition illustrate that the mean estimated speed difference between groups varied as a function of condition. Notably, interaction βs are positive for low angular frequencies and negative for high angular frequencies. This indicates that, at high angular frequencies the difference in speed between the autistic and non-autistic groups is larger than that which would be predicted from the main effects alone (and smaller than predicted at low angular frequencies).

Supplementary Table 2 Model Parameters for Jerk and Speed Mixed Models

	β estimate	SE	t statistic	DoF	p value	Lower CI	Upper CI
							**Jerk**
Intercept	5.25	0.11	47.82	1285	<.001***	5.03	5.46
Non-autistic	-0.20	0.11	-1.90	1285	.058	-0.41	0.01
Autistic	0.20	0.11	1.90	1285	.058	-0.01	0.41
2/33	-0.17	0.04	-4.22	1285	<.001***	-0.25	-0.09
2/5	-0.15	0.04	-4.08	1285	<.001***	-0.23	-0.08
4/5	-0.10	0.04	-2.85	1285	.004**	-0.18	-0.03
4/3	0.07	0.03	2.23	1285	.025*	0.01	0.14
2	0.33	0.03	10.24	1285	<.001***	0.27	0.40
3	0.08	0.04	2.07	1285	.039*	0.00	0.16
4	-0.06	0.04	-1.44	1285	0.149	-0.14	0.02
Non-autistic 2/33	0.10	0.04	2.60	1285	.009**	0.03	0.18
Non-autistic 2/5	0.17	0.04	4.55	1285	<.001***	0.10	0.25
Non-autistic 4/5	0.17	0.04	4.51	1285	<.001***	0.10	0.24
Non-autistic 4/3	0.09	0.03	2.74	1285	.006**	0.03	0.16
Non-autistic 2	-0.21	0.03	-6.39	1285	<.001***	-0.27	-0.14
Non-autistic 3	-0.18	0.04	-4.72	1285	<.001***	-0.26	-0.11
Non-autistic 4	-0.14	0.04	-3.28	1285	.001**	-0.22	-0.06
							**Speed**
Intercept	0.93	0.10	9.70	1285	<.001***	0.74	1.12
Non-autistic	-0.10	0.10	-1.08	1285	.282	-0.29	0.09
Autistic	0.10	0.10	1.08	1285	.282	-0.09	0.29
2/33	-0.09	0.02	-4.59	1285	<.001***	-0.13	-0.05
2/5	-0.23	0.02	-10.38	1285	<.001***	-0.27	-0.18
4/5	-0.27	0.02	-11.47	1285	<.001***	-0.31	-0.22
4/3	0.00	0.02	0.02	1285	0.981	-0.04	0.04
2	0.32	0.02	20.29	1285	<.001***	0.29	0.35
3	0.18	0.02	10.28	1285	<.001***	0.14	0.21
4	0.09	0.02	4.61	1285	<.001***	0.05	0.12
Non-autistic 2/33	0.06	0.02	2.97	1285	.003**	0.02	0.10
Non-autistic 2/5	0.10	0.02	4.38	1285	<.001***	0.05	0.14
Non-autistic 4/5	0.08	0.02	3.29	1285	.001**	0.03	0.12
Non-autistic 4/3	0.04	0.02	2.35	1285	.019*	0.01	0.08
Non-autistic 2	-0.10	0.02	-6.26	1285	<.001***	-0.13	-0.07
Non-autistic 3	-0.10	0.02	-5.90	1285	<.001***	-0.14	-0.07
Non-autistic 4	-0.07	0.02	-4.02	1285	<.001***	-0.11	-0.04

Note. Model equation: DV ~ Condition + Group + (Condition x Group) + (1|Trial) + (1|Participant), 'Link', 'log'. Statistics as produced by the Matlab fitglme function. *p < .05, **p < .01, ***p < .001.

Autistic participants decompose shapes into a greater number of submovements at higher angular frequencies

For submovements there was no significant main effect of group ($F(1,1285) = 3.50$, $p = .061$). However, there was a significant main effect of condition ($F(6, 1285) = 88.71$, $p < .001$) and a group x condition interaction ($F(6, 1285) = 6.31$, $p < .001$). Coefficients from a mixed effects model (submovements ~ Condition + Group + (Condition x Group) + (1|Trial) + (1|Participant)), can be seen in **Supplementary Table 3**. The main effect of group indicates that autistic participants decomposed movements into a greater number of submovements compared to non-autistic participants. The main effect of condition indicates that, compared to the grand mean, low angular frequency shapes were decomposed into more submovements whereas higher angular frequency shapes were decomposed into fewer submovements. The β coefficients (**Supplementary Table 3**) and **Supplementary Fig. 1** illustrate a U-shaped relationship such that the fewest submovements were produced for the elliptical shape.

β coefficients relating to the interaction between group and condition illustrate that the mean estimated difference in submovements between groups varies as a function of condition. Notably, this interaction term was only statistically significant for conditions 4/5, 2 and 4. Thus, for all conditions aside from 4/5, 2 and 4, mean submovements can be relatively accurately predicted from the combination of the main effect of condition, plus an additional adjustment for group (e.g., intercept + autistic + 2/33). However, 4/5, 2 and 4 do not show this typical pattern. For 4/5, the difference between the groups is smaller than the difference that would be predicted from the main effects (non-autistic 4/5 is positive, also see **Supplementary Fig. 1**). For conditions 2 and 4 the difference between the groups is greater than the difference that would be predicted from the main effects (non-autistic 2 and non-autistic 4 are negative, see **Supplementary Fig. 1**)

Supplementary Table 3 Model Parameters for Submovements Mixed Models

	β estimate	SE	t statistic	DoF	p value	Lower CI	Upper CI
							**Submovements**
Intercept	*3.73*	*0.06*	*63.76*	*1285*	*<.001****	*3.61*	*3.84*
Non-autistic	-0.11	0.06	-1.87	1285	.061	-0.22	0.01
Autistic	0.11	0.06	1.87	1285	.061	-0.01	0.22
2/33	0.50	0.03	15.91	1285	*<.001****	0.44	0.56
2/5	0.31	0.03	9.72	1285	*<.001****	0.24	0.37
4/5	0.11	0.03	3.51	1285	*<.001****	0.05	0.18
4/3	-0.12	0.03	-3.85	1285	*<.001****	-0.19	-0.06
2	-0.45	0.03	-13.88	1285	*<.001****	-0.51	-0.38
3	-0.21	0.03	-6.68	1285	*<.001****	-0.28	-0.15
4	-0.14	0.03	-4.18	1285	*<.001****	-0.20	-0.07
Non-autistic 2/33	-0.02	0.03	-0.63	1285	.528	-0.08	0.04
Non-autistic 2/5	0.05	0.03	1.59	1285	.111	-0.01	0.11
Non-autistic 4/5	0.14	0.03	4.27	1285	*<.001****	0.07	0.20
Non-autistic 4/3	0.06	0.03	1.82	1285	.069	0.00	0.12
Non-autistic 2	-0.11	0.03	-3.54	1285	*<.001****	-0.18	-0.05
Non-autistic 3	-0.03	0.03	-0.84	1285	.401	-0.09	0.04
Non-autistic 4	-0.08	0.03	-2.61	1285	.009**	-0.15	-0.02

Note. Model equation: DV ~ Condition + Group + (Condition x Group) + (1|Trial) + (1|Participant). Statistics as produced by the Matlab fitlme function. *p < .05, **p < .01, ***p < .001.

Supplementary Material 3

FFT of curvature confirms matched task compliance between groups

The FFT analysis was conducted in the curvature domain and revealed no significant differences between groups (see **Supplementary Fig. 2**). Given that this comparison of curvature values derived from the groups' drawings identified no clusters of statistical significance, our findings indicate that task compliance was matched between autistic and non-autistic individuals i.e., compared to non-autistic participants, those with autism did not draw more/less curvy trajectories.

Supplementary Figure 2 Amplitude Spectral Density Graph of Curvature. Amplitude spectral density of curvature for all angular frequency-defined conditions aligned to zero. Black line = autistic, Grey line = non-autistic, Red line = autistic - non-autistic.

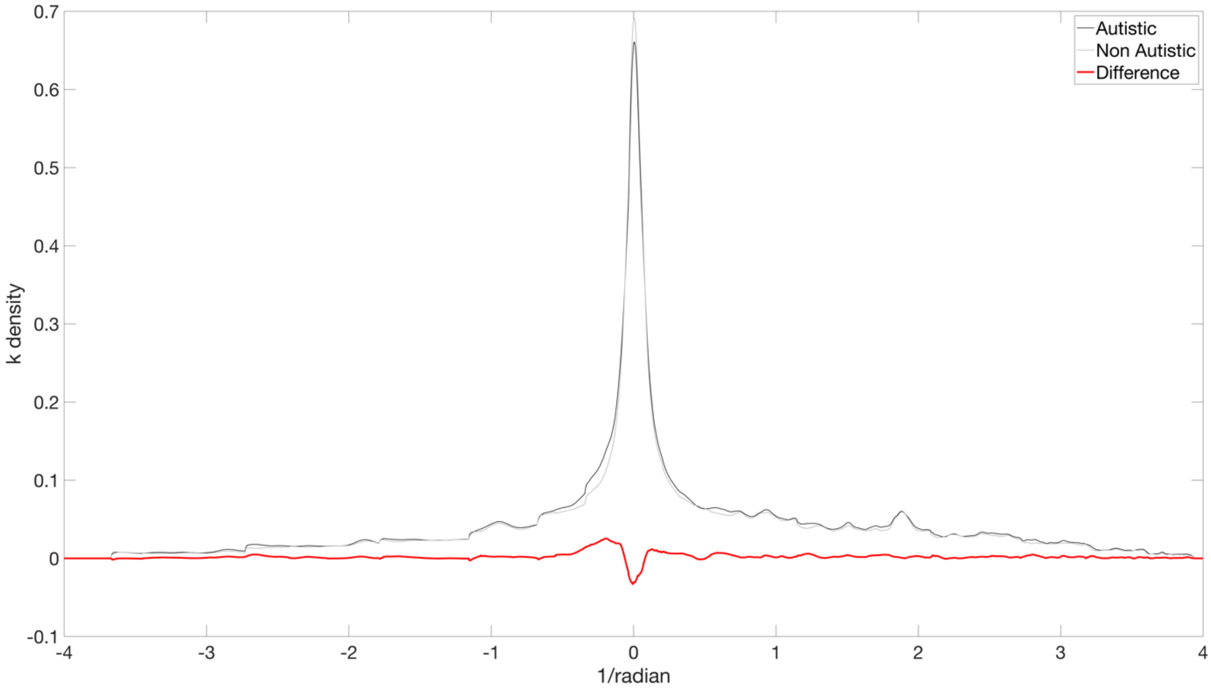

Supplement: Supplementary file 1 — Supplementary Material 1 [file 41598_2026_37067_MOESM1_ESM.pdf]
